# Studying the Complex Expression Dependences between Sets of Coexpressed Genes

**DOI:** 10.1155/2014/940821

**Published:** 2014-07-24

**Authors:** Mario Huerta, Oriol Casanova, Roberto Barchino, Jose Flores, Enrique Querol, Juan Cedano

**Affiliations:** ^1^Institut de Biotecnologia i Biomedicina, Universitat Autònoma de Barcelona, Bellaterra, 08193 Barcelona, Spain; ^2^Escola Tècnica Superior de Ingenieria, Universitat Autònoma de Barcelona, Bellaterra, 08193 Barcelona, Spain; ^3^Laboratorio de Inmunología, CENUR del Noroeste, Universidad de la República-Salto, Rivera 1350, 50000 Salto, Uruguay

## Abstract

Organisms simplify the orchestration of gene expression by coregulating genes whose products function together in the cell. The use of clustering methods to obtain sets of coexpressed genes from expression arrays is very common; nevertheless there are no appropriate tools to study the expression networks among these sets of coexpressed genes. The aim of the developed tools is to allow studying the complex expression dependences that exist between sets of coexpressed genes. For this purpose, we start detecting the nonlinear expression relationships between pairs of genes, plus the coexpressed genes. Next, we form networks among sets of coexpressed genes that maintain nonlinear expression dependences between all of them. The expression relationship between the sets of coexpressed genes is defined by the expression relationship between the *skeletons* of these sets, where this *skeleton* represents the coexpressed genes with a well-defined nonlinear expression relationship with the *skeleton* of the other sets. As a result, we can study the nonlinear expression relationships between a target gene and other sets of coexpressed genes, or start the study from the *skeleton* of the sets, to study the complex relationships of activation and deactivation between the sets of coexpressed genes that carry out the different cellular processes present in the expression experiments.

## 1. Introduction

Organisms have evolved to vary internal and external cell environments by carefully controlling the abundance and activity of these proteins to suit their conditions. To simplify this task, genes whose products function together are often under common regulatory control. This regulatory control is such that these genes are coordinately expressed under the appropriate conditions. The experimental observation that a set of genes is coexpressed frequently implies that the genes share a biological function and are under common regulatory control [1]. These regulators that govern the expression of sets of coexpressed genes that carry out the appropriate cell functions are also regulated and synchronized among them. Nevertheless, the regulation and synchronization among the regulatory mechanisms is much more complex. These regulatory mechanisms are not directly regulated by the other regulatory mechanisms, but by the coexpressed genes product of their activation cascade. These coexpressed genes switch from the inhibition to the allowance of a regulatory mechanism depending on if they reach or lose certain expression levels. Furthermore, these regulatory mechanisms are multiregulated. The final activation or deactivation of a regulatory process will depend not only on internal and external factors to the cell but also on the expression level of a set of coexpressed genes. For this reason, to study the synchronization and regulation among the regulatory mechanisms based on gene expression is really difficult. Furthermore, many proteins have multiple roles in the cell and act with distinct sets of cooperating proteins to fulfil each role. The genes that synthesize these proteins are therefore coexpressed with different sets of genes, each one governed by a distinct regulatory mechanism, in response to the varying demands of the cell. The experimental conditions will determine the regulatory mechanism activated in each case and thus the set of coexpressed genes that will be activated. An increased number of different experimental conditions for the same genes will provide less genes in each set of coexpressed genes, more different sets, and higher alternation of the activation and deactivation of the coexpressed genes. But irrespective of the conditions, how can we study the effect of their activation and deactivation on the rest of sets of coexpressed genes?

Microarray technology, as well as the new techniques of next generation sequencing (NGS), allows us to obtain large size gene expression arrays [2]. These gene expression arrays contain the expression levels of thousands of genes for tens of sample conditions. The usual analyses of these data are focused on the differentially expressed genes [3] as well as on the use of clustering methods to obtain the sets of coexpressed genes. It is of vital importance to detect these clusters of coexpressed genes, among other reasons, because, as mentioned before, these clusters of coexpressed genes carry out the different cellular functions [1]. There are powerful coexpression analysis tools for this purpose [4, 5]. As expression arrays allow simultaneous analyses of thousands of genes, we can study genes responsible for very diverse cellular functions. Therefore, it makes it easier for the researcher to understand the cellular behaviour in the performed experiments from a holistic point of view, that is, involving the largest number of cellular processes possible. Without this holistic point of view it is very difficult to deal with the multiple functions, phenotypes, or states of the living beings, in which a large amount of genes are collaborating. This holistic point of view can be useful to characterize phenotypes previously unknown, like for instance the description of the “fish fever” in zebrafish [6]. Nevertheless, even though clustering methods allow us to obtain coexpressed genes and thus differentiate diverse cellular processes, clustering methods explain very little of the expression relationships between the different sets of coexpressed genes. As a consequence, the researcher is constrained to study each one of the coexpressed gene sets individually, losing much of the potential that the technologies for obtaining gene expression arrays offer.

Current statistical technologies allow us to study inclusion relationships between clusters of coexpressed genes, that is, to study which clusters of coexpressed genes would be more correlated and which others would be more uncorrelated [7]. But they do not go much beyond that. The main obstacle to the tools that attempt to study the regulation of coexpressed genes is that the low number of copies of the regulatory genes impedes the correct capture of their expression by technologies to obtain gene expression. Furthermore, the coexpression of genes is strongly linked to the chromatin structure, especially in multicellular organisms. This chromatin structure depends on a complex network of cellular signalling and posttranscriptional modifications of proteins (phosphorylation, acetylation, ubiquitination, etc.). So, even having detected the regulatory genes, we would have an incomplete puzzle. All of this makes it enormously difficult for the tools to be able to obtain information about the regulation among the sets of coexpressed genes and the processes they carry out. Then, without these regulatory elements, how can we describe a regulatory network among the processes performed by the sets of coexpressed genes? Based on the dependences between the gene expressions product of this complex regulation. Furthermore, these final genes present expression ranges wide enough so the fluctuations of their expression dependences can be analysed.

With the developed tools, we expect to detect the complex expression dependences between the different sets of coexpressed genes, synthesize them, and make them easier for the researcher to interpret. With this purpose we will provide the researcher with networks that show the expression dependences between all the coexpressed gene sets of the network. In this way, the researcher will be able to study the alternation or synchronism among all these sets of coexpressed genes.

Our methodology is based on the following three principles. First, the interdependence between sets of coexpressed genes cannot be described by linear expression relationships. Second, if two genes maintain an expression relationship with a certain type of curve, the genes coexpressed with these two genes maintain expression relationships of the same type between them. Third, the curve type of the intergroup expression relationships will describe the dependence of activation and deactivation between these sets of coexpressed genes. Thus, the strategy proposed here is focused on the detection of nonlinear expression relationships between sets of coexpressed genes.

Activation and deactivation dependences between sets of coexpressed genes can be very complex; some of the most common ones are those in which a set of coexpressed genes act as a trigger of another set of coexpressed genes; the case of antagonist processes, where the coexpressed gene set that carries out each process needs to be totally deactivated so the other set can express; or sets of coexpressed genes that activate or deactivate another set of coexpressed genes when losing their basal values of expression. In any case, the system does not anticipate any type of expression relationship. Since the system is able to recognize curves of very different shapes, it can process unknown activation and deactivation relationships as reliably as when processing the best known relationships.

There are multiple works that highlight the relevance of the analysis of nonlinear expression relationships [8–10]. In the last cited work [10] the difference of considering the nonlinear relationships with respect to considering only the linear relationships can be observed very clearly.

As it is shown in the mentioned paper [10], nonlinear expression relationships allow detecting new hubs in gene networks, because they allow relating genes by complex expression relationships and to discover new relationships that otherwise would not be possible to detect. Now, the next step in the analysis based on nonlinear expression relationships is to deepen in the study of complex expression dependences between sets of coexpressed genes. In order to perform this task we need to detect the types of curve of the expression relationships, since the type of curve describes the type of relationship between the sets of coexpressed genes and thus between the processes that these sets of genes carry out.

The ultimate goal of our approach is that researchers are able to know the networks of processes hidden in their experimental data, as well as the activation and deactivation relationships between all of these processes. Furthermore, if the researcher is particularly interested in specific genes, the system will allow him/her to study the way the expression of a gene activates and deactivates different processes from the process that this gene, and those genes coexpressed with it, carry out.

## 2. Methodology

### 2.1. The Suitable Expression Data to Be Analysed

The appropriate data to be analysed by our methodology must come from large sample series (i.e., expression matrices with a high number of sample conditions). This large sample series will not consist of repetitions of the same sample condition; on the contrary, it must include the highest number of different sample conditions. A sample series with few experiments or with repetitions of the same experiment will not allow detecting coexpressed genes and even less to detect complex expression relationships. Note that de-noise, normalization, and similar procedures should be considered before using our tools.

The examples provided in the paper and supplementary materials use the data from* AT*_matrix. This matrix is the correlation between survival (*A*_matrix) and expression (*T*_matrix).* A*_matrix contains the growth inhibitory activities of 118 compounds tested on 60 tumour cell lines. This compound set includes most of the drugs currently in clinical use for tumour treatment. The microarray data (*T*_matrix) reflect the level of expression of 1376 genes, plus 40 individually assessed targets (proteins) and 40 other targets in the previous 60 tumour cell lines.* AT*_matrix links both matrices using the 60 tumour cell lines as a sample space to generate a correlation matrix of 1416 rows (genes and targets) by 118 columns (substances) [13]. These expression data were chosen because they cover a wide range of phenotypes shared by many different human tumour tissues.

### 2.2. PCOP

The mathematics behind this system uses the principal curves of oriented points (PCOP) calculation [10] to describe the expression relationships inner pattern. The principal curves is a nonlinear and nonvariable-dependent analysis technique, which is very suitable for the nonlinear analysis of expression relationships [14]. PCOP is defined by the generalization, at local level, of the following principal-component property: for a normal multivariable distribution *X*, if *X* is projected over a hyperplane, the total variance of the projection is minimised when the hyperplane is orthogonal to the first principal component. In this way, a principal oriented point (POP) is found for each local area, and the curve that goes throughout all of the POPs is the PCOP. The PCOP method provides the uncorrelation factor *f*. The *f* calculation considers not only the data dispersion around the curve, but also how well the curve pattern describes the morphology of the data cloud for any continuous curve type [10]. Thus, this *f* value provides an excellent measure for the nonlinear correlations.

### 2.3. Genes Coexpressed with a Pair of Genes with a Nonlinear Expression Relationship between Them

All the nonlinear expression relationships are detected for each gene of the expression array. These nonlinear expression relationships are classified by the type of curve. The* curvature points* of the PCOP are used to identify and classify the nonlinear expression relationships.* Curvature points* are those POPs in the PCOP in which a change in slope occurs. The detection of* curvature points* in expression relationships identifies the nonlinear expression relationships. The type of curve is described by the function of the curve: *y* = *e* ^*x*^, *y* = −*e* ^*x*^, *y* = −ln⁡(*x*), *y* = *x* ^2^, *y* = −*x* ^2^, *y* = *x* ^3^, 1 = *x* ^2^ + *y* ^2^, and so on. The genes coexpressed with each gene are also detected (none* curvature points* are detected in the PCOP of two coexpressed genes). This will allow us to study the nonlinear expression relationships between a user's gene of interest and different sets of coexpressed genes.

The correlation degree provided by the PCOP calculus is what guarantees us that the linear expression relationships (coexpressed genes) as well as the nonlinear expression relationships (intergroup expression relationships) are not a product of chance and have a biological meaning. For this reason, we require a high correlation degree for the linear expression relationships as well as for the nonlinear expression relationships. We are also restrictive in the classification of the expression relationships as linear expression relationships and the consequent consideration of two genes as coexpressed genes. Even a small curvature in the relationship of two coexpressed genes can cause a diversity in the typology of the expression relationships of these two genes with the genes of another set of coexpressed genes, more concretely, being A and B, two coexpressed genes whose linear expression relationship has a small curvature, and being C, a set of coexpressed genes that maintain nonlinear expression relationships with A and B. The expression relationships of gene A with set C may describe a different typology with respect to the expression relationships of gene B with set C.

### 2.4. Cliques of Nonlinear Expression Relationships between Genes

A clique in an undirected graph is a subset of its vertices such that every two vertices in the subset are connected by an edge. If we consider a graph of all the nonlinear expression relationships with a high correlation, we obtain its cliques. These cliques will not relate sets of coexpressed genes yet, but genes individually. Nevertheless, as the cliques are not relating pairs of genes but several genes in a network of nonlinear expression relationships, the cliques will be the seed to relate the sets of coexpressed genes between all of them. The genes of a clique must be at least three and they must maintain nonlinear expression relationships between all the genes of the clique.

### 2.5. Pairs of Isomorphic and Linear Cliques of Nonlinear Expression Relationships

Once the cliques are detected, they are grouped in pairs by relating genes that belong to the same set of coexpressed genes.

The cliques that will form each pair will meet two conditions.

1. Each one of the genes of a clique will be coexpressed with a different gene of the other clique, forming pairs of coexpressed genes.
2. The type of curve that relates two pairs of coexpressed genes will be the same in both cliques.

This provides us with pairs of cliques. Each gene of a clique will be coexpressed with a different gene of the other clique forming pairs of coexpressed genes. Then, the expression relationships that relate genes from different pairs of coexpressed genes will maintain the same type of curve for the two genes of the pair.

### 2.6. Cliques of Isomorphic and Linear Cliques of Nonlinear Expression Relationships among Genes

On the previous section we obtained the nonlinear expression relationships between pairs of coexpressed genes. Now, we obtain sets of coexpressed genes that maintain nonlinear expression relationships between them by grouping these pairs of coexpressed genes into sets of coexpressed genes. If we consider a graph where the vertices are the cliques of nonlinear relationships between genes and the edges link the pairs of linear isomorphic cliques, now we will calculate the cliques of this new graph obtaining the cliques of cliques. Thereby we obtain the* skeleton* of each set of coexpressed genes and the nonlinear expression relationships between the* skeletons. *The different networks among sets of coexpressed genes will be formed from the relationships between the* skeletons* of each set of coexpressed genes.

The genes of the* skeleton* of a set of coexpressed genes are those genes of the set that maintain a high-correlated nonlinear expression relationship with the* skeleton* of the other sets. The genes of the set of coexpressed genes that are not part of the* skeleton* will be coexpressed with the genes of the* skeleton*. These genes coexpressed with the* skeleton* will maintain the same type of nonlinear expression relationship with the other sets of coexpressed genes the closer they are to a *y* = *x* relationship with respect to the genes of the* skeleton*. Deformations of this *y* = *x* relationship between the genes of the set and its* skeleton* genes will produce a distortion of the expected curve. A higher variance in the coexpression with respect to the* skeleton* genes will also imply a higher variability in the expected type of curve.

The higher the number of genes of the* skeleton *is, the more representative the processes carried out by the coexpressed gene sets are. Thanks to the second condition of the linear-isomorphic-cliques definition we can make sure that the relationships between the genes of the* skeleton* of different sets of coexpressed genes maintain the same type of curve for all the genes of the* skeleton*. In the same way, we can also make sure that the genes coexpressed with the* skeleton* maintain expression relationships also of the same type (although it will depend on the correlation degree and how close to *y* = *x* the coexpressed gene is).

The correlation degree to consider the expression relationships coexpressed enough will depend on the number of genes of the expression array. It is useful for small expression matrices, where nonlinear expression relationships between sets of coexpressed genes can be detected although these expression relationships have high entropy. The aim is to always detect enough nonlinear expression relationships to be able to find the* skeletons* that relate the sets of coexpressed genes.

The threshold to consider an expression relationship as linear or nonlinear will also depend on the number of genes, being (this threshold) more restrictive for the linear ones in matrices with less genes. Thus, large sets of coexpressed genes with very sharp curves between them can be formed for large expression matrices, whereas smaller sets of coexpressed genes, as well as more subtle nonlinear expression relationships between the sets, will be considered for small matrices.

The expression relationships have been filtered by the uncorrelation factor provided by the PCOP calculation to be considered correlated enough [10]. The threshold formula is

(1) $$\begin{array}{c} 0.12\times \frac{1600}{\text{num}\;\text{genes}}-{\left(\frac{\text{num}\;\text{genes}}{40000}\right)}^{18}. \end{array}$$

The threshold formula for the curvature to consider whether the relationships are linear or nonlinear is

(2) $$\begin{array}{c} 160-\left(\frac{\left(15.0/20000\right)+\left(14.0/18400\right)}{2\times \text{num}\;\text{genes}}\right). \end{array}$$

However, a formula that depends only on the number of genes is not enough to guarantee the quality of the analysis extracted from the data because the data correspond to very different experiments with different nature. The diverse nature of the experiments is a qualitative variable that cannot be quantified with a formula. For this reason the correlation threshold calculation uses an online correction: from the relationships already analysed and the number of relationships pending to be analysed, the system makes an estimation of the number of relationships that would finally pass the threshold. From the calculated estimation, the system modifies automatically the threshold.

A higher number of genes in the expression array increase the number of coexpressed genes and nonlinear expression relationships, which facilitates finding* skeletons*. But in any case, the number of expression relationships with high correlation, as well as the number of linear expression relationships with respect to the nonlinear ones, will always depend on the nature of the experiments of the sample series.

## 3. Results and Discussion

The system allows to study sets of coexpressed genes that maintain nonlinear expression relationships among them, as well as to study the nonlinear expression relationships that a concrete gene of interest maintains with different sets of coexpressed genes. This can be studied for this target gene as well as for the genes coexpressed with it. There have been found 4573 nonlinear relationships and 20269 pairs of coexpressed genes (all highly correlated) from the microarray of 1416 genes used in the examples.

### 3.1. Searching for the Complex Expression Relationships between a Target Gene and Sets of Coexpressed Genes

The study of the expression relationships between sets of coexpressed genes can start from the researcher's genes of interest. All the nonlinear expression relationships that a gene of interest maintains with different sets of coexpressed genes will be shown. These relationships will be shown classified by curve type, because each curve type implies a different activation/deactivation relationship. There will be shown only the nonlinear expression relationships that maintain a sufficient correlation degree.

**Figure 1:**
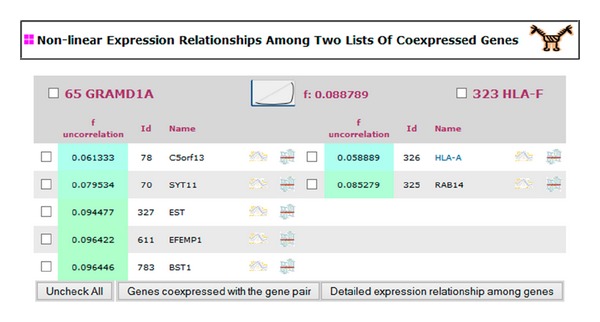
This view shows a nonlinear expression relationship where a researcher's gene of interest participates. The gene of interest is displayed on the top of the view on the left side. The column on the left side displays the genes coexpressed with the gene of interest, while the column on the right displays a set of coexpressed genes that maintain a nonlinear expression relationship with the gene of interest. The coexpressed genes are ordered by their correlation degree with their respective gene at the top (the *f* value obtained by the PCOP calculation). The icon shows the curve type of the nonlinear expression relationship. Each curve type implies a different expression dependence: mutual exclusion, trigger, double trigger, and so on. All the expression relationships relating genes from the two sets of coexpressed genes should be of the type shown by the icon. By selecting genes from the two sets, their expression relationship can be studied in detail in a new interface (Figure 3).

We will study the activation and deactivation relationship between our gene of interest and different sets of coexpressed genes starting from these high-correlated nonlinear expression relationships. Two lists of coexpressed genes will be shown in a new view for each high-correlated nonlinear expression relationship of the gene of interest (Figure 1). The first list will show the genes coexpressed with the gene of interest. The second one will show the genes coexpressed with the gene that maintains the high-correlated expression relationship with the gene of interest. Then, the user can study the expression relationships between the two sets of coexpressed genes. The user can select genes from both lists of coexpressed genes to study in detail their expression relationship using a different interface [11, 12] (Figure 3). The first list of coexpressed genes is ordered by their correlation degree with the gene of interest and the second list is ordered by their correlation with respect to the gene nonlinearly related to the gene of interest.

An icon shows the type of nonlinear relationship between the two main genes and, by extension, between the two sets of coexpressed genes. The curve type is very important, since it determines the role of the genes in each expression dependence.

### 3.2. The Curve Type Indicates the Type of Activation and Deactivation Relationship between Sets of Coexpressed Genes

The system obtains the inner pattern of the curve for any type of expression relationship and classifies it. The only requirement is that the data cloud must be continuous. A *y* = *e* ^*x*^ relationship will provide an activation relationship between a set of genes and the other set; in other words, the first set of coexpressed genes must overexpress so the second set starts to express. In a *y* = −*e* ^*x*^ relationship, instead of an activation of the second set, there will be a deactivation. A *y* = −ln⁡⁡(*x*) relationship indicates a mutual-exclusion dependence; that is, one of the two sets of genes must be deactivated so the other set of coexpressed genes expresses. Note that these types of relationships are different from the positive and inverse coexpression relationships. This difference is precisely what allows us to detect different sets of coexpressed genes as well as the complex expression dependences between them. On the other hand, a *y* = −*e* ^*x*^ relationship, a *y* = −*x* relationship, and a *y* = −ln⁡(*x*) relationship explain completely different activation/deactivation dependences.

A *y* = *x* ^2^ relationship would indicate a deactivation of the second set of coexpressed genes by the overexpression as well as the underexpression of the first set. A *y* = −*x* ^2^ relationship would indicate an activation of the second set of genes, for the overexpression as well as the underexpression of the first set. Whereas in the relationships of type |*e* ^*x*^|, the overexpression of the set of coexpressed genes affects the other set. In the relationships of type |*x* ^2^|, the overexpression as well as the underexpression of the genes has an inhibitory or activatory effect on the other set of coexpressed genes.

Other relationships, such as those of type *y* = *x* ^3^ or 1 = *x* ^2^ + *y* ^2^, will indicate other complex expression dependences between different sets of coexpressed genes. Real examples of each type of curve are shown in the supplementary material available at http://platypus.uab.es/nlnet.

As pointed out in the introduction, one of the three principles of our methodology is as follows. The type of curve between two genes is also maintained between the genes coexpressed with each one of them. Let us see an example: HLA genes are indicative of cell maturation marking the cell so it is recognised by the immune system [15]. HLA genes mark the cell making possible the inflammation of the tissue and the activation of the immune system. GRAMD1A is not a well-known membrane receptor that inhibits programmed cell death and it is linked to disease resistance [16]. HLA-F and GRAMD1A maintain a nonlinear expression relationship of type *y* = *e* ^*x*^ (Figure 3(a)). This points out that, possibly, the function associated with GRAMD1A can only be performed once the cell is marked by HLA genes. HLA-F and HLA-A are coexpressed genes (Figure 3(b)), and GRAMD1A is coexpressed with NREP (Figure 3(c)). Thus, HLA-A and NREP will also maintain a nonlinear expression relationship of type *y* = *e* ^*x*^ (Figure 3(d)). C5orf13 (NREP) expression is linked to hypertrophic scar [17]. This points out that hypertrophic scar and the function associated with NREP can only be performed once the cell is marked by HLA genes [18]. Even though the relationship of these genes with hypertrophic scar was already known [17, 18], there was no knowledge about how it was regulated.

Hypertrophic scarring (HS) is a result of increased fibrogenesis, which is thought to be caused by an exaggerated inflammatory response [19–21]. There is a clear association between specific HLA alleles and cutaneous fibrosis. Specific examples of cutaneous fibrosis include hypertrophic scars (HS) among others [18].

The relation of HLA and hypertrophic scar is already documented, but using our tool we found that this relation is mediated by NREP, because HLA genes must be overexpressed to activate NREP (a gene directly linked to HS [17]).

In this way, it is valuable that even though the technologies to obtain gene-expression arrays do not capture regulatory genes because of the low variability in their gene expression, these technologies do allow studying the regulation between processes through the genes that perform these processes (coexpressed genes that result from the activation cascade started by regulatory genes). This is because these final genes do maintain wide enough expression ranges, which allows our high-throughput tool to analyse the expression dependence between the sets of coexpressed genes.

### 3.3. Studying the Complex Expression Relationships between Sets of Coexpressed Genes

The different networks of nonlinear expression relationships among sets of coexpressed genes are classified by the number of sets and the curve types of the expression relationships between the sets. Once a network type is selected, the networks found in the expression matrix that maintain this pattern in the intergroup expression relationships are displayed.

**Figure 2:**
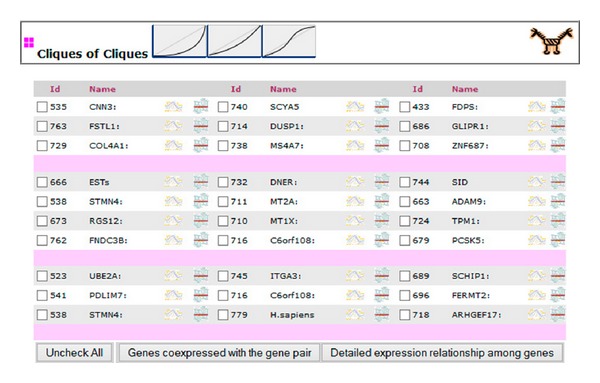
This view shows networks of concrete types of nonlinear expression relationships between sets of coexpressed genes. The icons at the top show the curve-type pattern of the networks listed. The networks will always form a complete graph. The pink line separates the networks found for the curve-type pattern. The columns contain the genes of the* skeleton* of each set of coexpressed genes. The genes of the different* skeletons* can be selected to study their expression relationship in detail [11, 12] (Figure 3). The genes of the different* skeletons* can be selected to study the expression relationships between the rest of coexpressed genes of the two sets, opening the view of Figure 1 for the two* skeleton* genes.

In the view that shows the networks (Figure 2), the genes that belong to the* skeleton* of each set of coexpressed genes are displayed in different columns. Each column displays the genes of the* skeleton* of each set of coexpressed genes. By selecting genes from the different* skeletons*, the expression relationships between them can be studied. Starting from the genes of the* skeleton*, the expression relationships between the rest of coexpressed genes of the sets can also be studied. By selecting one gene from the* skeleton* of two different sets of coexpressed genes, the genes coexpressed with each one of the genes of the two* skeletons* will be shown (in the way shown in Figure 1). All of them should maintain the same type of nonlinear expression relationship between them. These listed genes can also be selected to study their nonlinear expression relationships in detail [11, 12]. In this way, it can be studied whether the genes coexpressed with the* skeleton* maintain the type of curve or whether it is distorted or lost. The genes coexpressed with the genes of the* skeleton* of the two sets appear ordered by their correlation degree with the gene of the* skeleton* of each set. The higher the correlation between a coexpressed gene and the gene of its* skeleton *is, the lower the variations of the curve types between this gene and the genes of the other set with respect to the curve type between the genes of both* skeletons *are.

**Figure 3:**
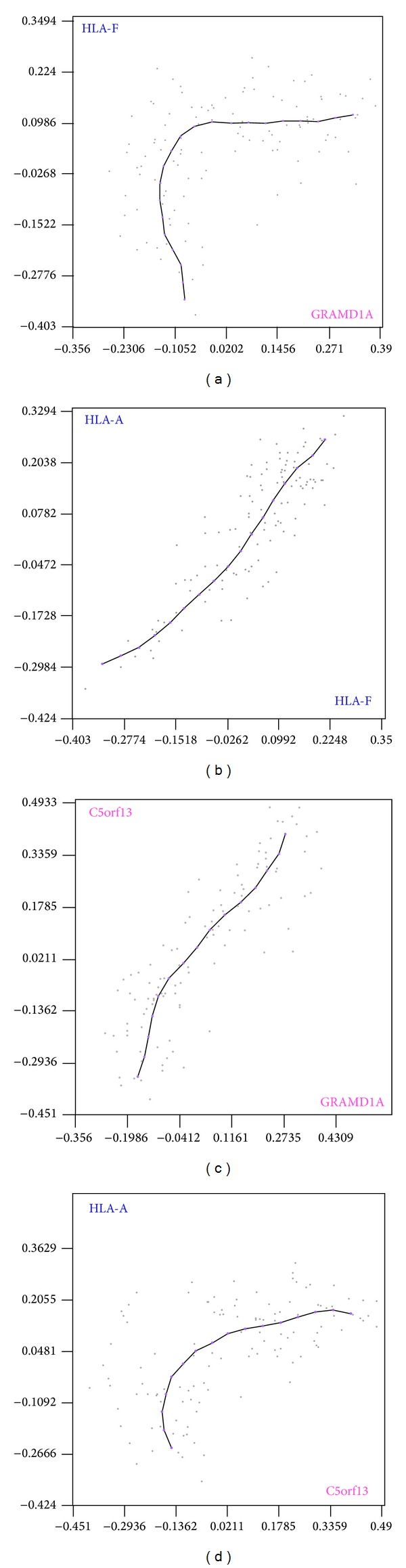
Four expression relationships are shown. The different sample conditions of the expression matrix (the sample series) constitute the data cloud. The PCOP describes the expression-relationship inner pattern. (b) and (c) show coexpressed genes. HLA-A and HLA-F are coexpressed genes (b) and GRAMD1A and NREP are also coexpressed genes (c). (a) and (d) show nonlinear expression relationships of *y* = *e* ^*x*^ type, an activation relationship. Since HLA-F and GRAMD1A maintain a nonlinear expression relationship of type *y* = *e* ^*x*^ (a), HLA-F is coexpressed with HLA-A (b), and GRAMD1A is coexpressed with NREP (c), therefore HLA-A and NREP (C5orf13) maintain a nonlinear expression relationship of the same type (d). HLA-A and GRAMD1A would also maintain a nonlinear relationship of type *y* = *e* ^*x*^, and HLA-F and NREP would maintain a nonlinear relationship of the same type. This is the key point of our approach: all the nonlinear expression relationships that relate genes from two sets of coexpressed genes will have the same type of curve.

In Figure 3 we can see the key point of our approach: all the nonlinear expression relationships that relate genes from two sets of coexpressed genes will describe the same type of curve. In this way we can start the analyses from the relationships between all the pairs of genes of the microarray, but we construct the relationships between the sets of coexpressed genes from the* skeletons*. Since each set carries out a different cellular process, using our tool we can study the relationships between these independent cellular processes.

## 4. Conclusions

To respond to diverse and frequently changing conditions, cells must precisely mediate the synthesis and function of the proteins in the cell. This is controlled in part by the overall genomic expression program that results from the combined action of different regulatory factors, each of which responds to specific extra- and intracellular signals. These regulators govern the expression of sets of coexpressed genes that perform the appropriate cell functions. The variations in the expression of these coexpressed genes can be captured by high-throughput technologies to obtain gene expression arrays. In this way, the researcher is able to know which processes are carried out in the conditions he/she wishes to study, by knowing the different genes coexpressed in them. But what if the researcher wishes to know more? What if he/she wishes to know which relations have those different processes between them? In the case of working with large sample series, how do we know how these processes are activating or deactivating and activating again among them? If the researcher suspects that certain target genes can be a therapeutic target, how can he/she know the effect of their expression on the rest of the processes that this target gene does not belong, since it expresses with a different set of coexpressed genes? To know this could be implied from discovering unknown side effects to finding new ways to manipulate the expression of this gene.

In order to solve all these issues, we perform our high-throughput analysis. We obtain the coexpressed genes and the high-correlated nonlinear expression relationships and from them we obtain cliques (complete graphs) between coexpressed gene sets that maintain nonlinear expression relationships between them. In these networks, all the sets of coexpressed genes maintain a nonlinear expression relationship with each and every one of the other sets of coexpressed genes of the network. So, anytime, you know how to move from one process to another, passing by any other intermediate process. As a result, multiple networks are provided by a dynamic system that allows detecting sets of coexpressed genes related between them by complex activation dependences. The networks found will always depend on the analysed microarray. Large enough sample series and wide enough gene expression ranges facilitate the detection of coexpressed genes as well as the detection of nonlinear expression relationships between them. It is important to note that to detect a nonlinear expression dependence between two sets of coexpressed genes, this nonlinear expression dependence must exist, even though it affects only two small sets of coexpressed genes. In case these dependences exist, the developed tools allow to obtain very relevant information for the researcher since it makes possible to observe how the sets of coexpressed genes of his/her experiment interact between all of them. We think this approach could be a useful complement to other computational methods commonly used to analyse gene expression data.

As we present in the introduction, the expression dependences between sets of coexpressed genes, as well as between the processes these sets of coexpressed genes carry out, would never be linear. This is why new tools like the presented one are necessary.
